# A case report of ventricular dysfunction post pericardiocentesis: stress cardiomyopathy or pericardial decompression syndrome?

**DOI:** 10.1186/s12947-015-0026-3

**Published:** 2015-07-16

**Authors:** Chadi Ayoub, Michael Chang, Leonard Kritharides

**Affiliations:** Department of Cardiology, Concord Repatriation General Hospital, Concord, 2139 NSW Australia; The University of Sydney, Sydney, NSW Australia

**Keywords:** Pericardiocentesis, Stress cardiomyopathy, Pericardial decompression syndrome, Ventricular dysfunction, Echocardiography

## Abstract

We report a case of transient biventricular dysfunction post therapeutic pericardiocentesis, with classic features of stress cardiomyopathy (SCM). In our patient, the clinical and echocardiographic features were more in keeping with Takotsubo-type SCM than pericardial decompression syndrome (PDS). Our case is instructive in challenging our understanding of the aetiology of LV dysfunction complicating pericardiocentesis, and in highlighting the importance of careful clinical evaluation (altered heart rate and dyspnoea) in suspecting acute LV dysfunction after initial clinical improvement with pericardial aspiration.

**Electronic supplementary material:**

The online version of this article (doi:10.1186/s12947-015-0026-3) contains supplementary material, which is available to authorized users.

## Background

We report a case of reversible biventricular dysfunction following successful pericardiocentesis with classic features of stress or “Takotsubo” cardiomyopathy (SCM). Reports of SCM after pericardiocentesis are rare [1], as distinct from so-called pericardial decompression syndrome (PDS) which encompasses a spectrum of features of cardiac decompensation after large volume pericardiocentesis, including pulmonary oedema, adult respiratory distress syndrome, severe bi-ventricular failure and cardiogenic shock [2]. Our case is instructive in challenging our understanding of the aetiology of LV dysfunction complicating pericardiocentesis, and in highlighting the importance of careful clinical observations (heart rate and dyspnoea) in suspecting acute LV dysfunction after initial clinical improvement with pericardiocentesis.

## Case report

A 62-year-old male presented with progressive dyspnoea for 10 days. He had a background of stage IV metastatic non-small lung carcinoma treated for 6 months with non-cardiotoxic chemotherapy (carboplatin and gemcitabine), and recently commenced on target therapy (Erlotinib). Clinical examination revealed signs consistent with cardiac tamponade, including significant pulsus paradoxus, tachycardia (heart rate 101), tachypnea (respiratory rate 25), elevated jugular venous pressure and muffled heart sounds. He was normotensive at 130/90mmHg. The patient was extremely anxious and spontaneously expressed concern about his imminent death.

**Fig. 1:**
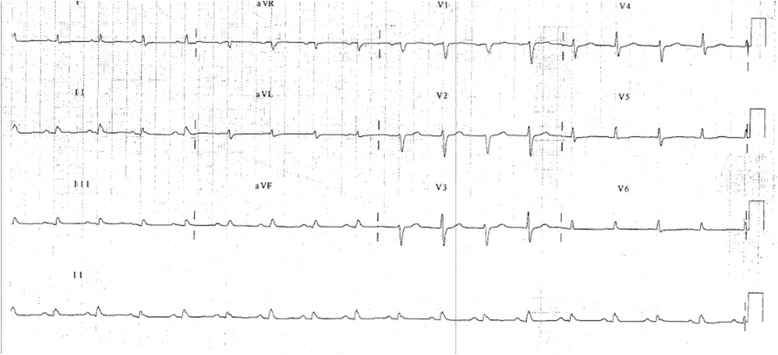
ECG on first presentation with tamponade demonstrating reduced voltage and electrical alternans

**Fig. 2:**
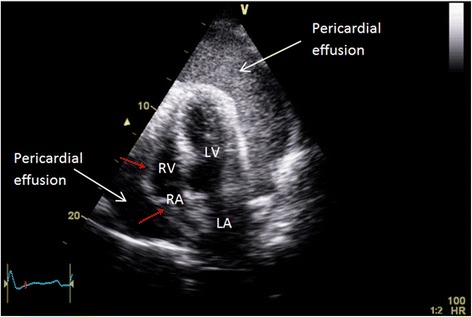
Apical four chamber view on initial presenation demonstating large pericardial effusion with tamponade causing compression of right heart chambers (red arrows). LV function was normal prior to pericardiocentesis

**Fig. 3:**
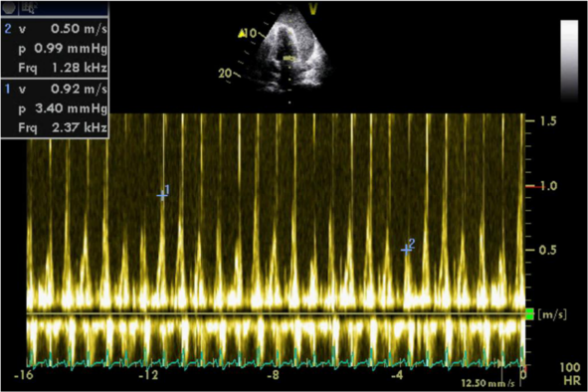
Transmitral inflow traces showing signicicant respiratory phase variation, consistant with tamponade on first presentation

His electrocardiogram (ECG) (Fig. 1) demonstrated electrical alternans and bedside transthoracic echocardiography (TTE) revealed a large pericardial effusion with features of cardiac tamponade, including diastolic compression of both right atrium and ventricle (Fig. 2, Additional file 1: Video 1 and Additional file 2: Video 2) and large mitral inflow variation (Fig. 3). Urgent pericardiocentesis was performed with a restricted aspiration of only 600 ml drained initially over the first hour, and a total drainage of 1.8 l of heavily blood-stained pericardial fluid over 36 h. During initial aspiration of pericardial fluid there was immediate symptomatic relief and haemodynamic improvement (heart rate [HR] decreased to 80/min, respiratory rate [RR] decreased to 15 breaths/min and BP increased to 150/70 mmHg).

**Fig. 4:**
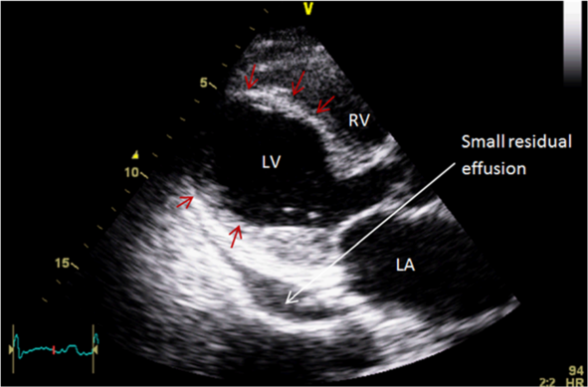
Parasternal long view post pericardiocentesis demonstrating apical ballooning (red arrows) as a result of apical and peri-apical akinesis

**Fig. 5:**
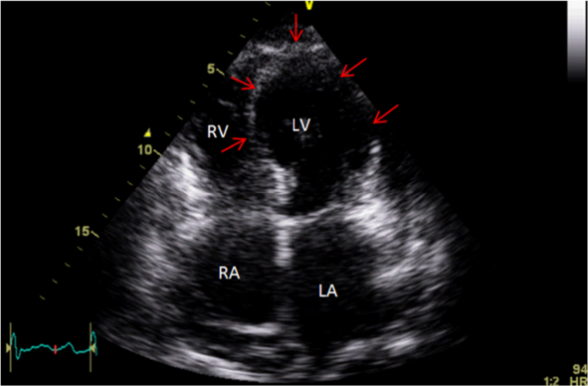
Apical four chamber view post pericardiocentesis demonstrating apical ballooning (red arrows) as a result of apical and peri-apical akinesis

**Fig. 6:**
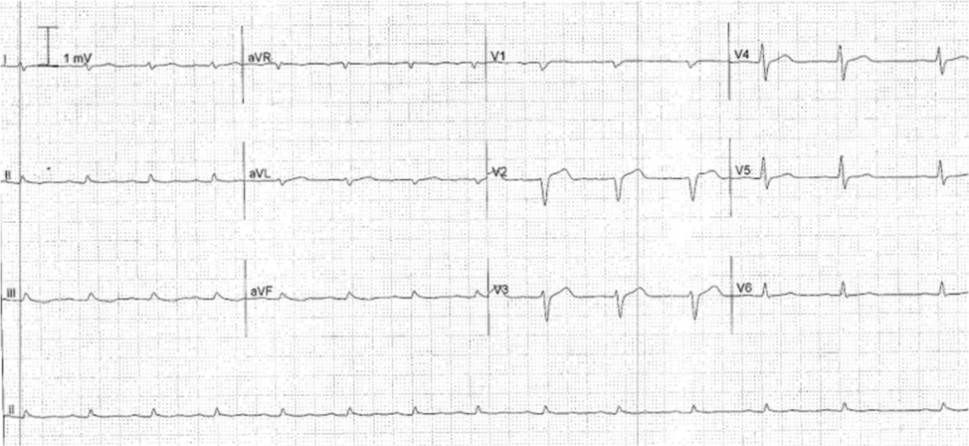
ECG after the chest discomfort following pericardiocentesis showing resolution of electrical alternans, and loss of R waves in V1 and V2

Overnight (9 h post procedure) the patient developed chest discomfort, dyspnea, tachycardia (HR 110) and tachypnoea (RR 24). TTE the next morning showed no re-accumulation of pericardial fluid, but detected new severe impairment in function of both ventricles, with akinesis of the apex and peri-apical region (Figs. 4 and 5, Additional file 3: Video 3 and Additional file 4: Video 4). Biomarkers demonstrated a rise in highly sensitive troponin from 8 to 224ng/L, but creatinine kinase did not rise significantly (107 to 116U/L). ECG after chest pain demonstrated resolution of the electrical alternans, with new loss of R waves in the anterior leads (Fig. 6).

**Fig. 7:**
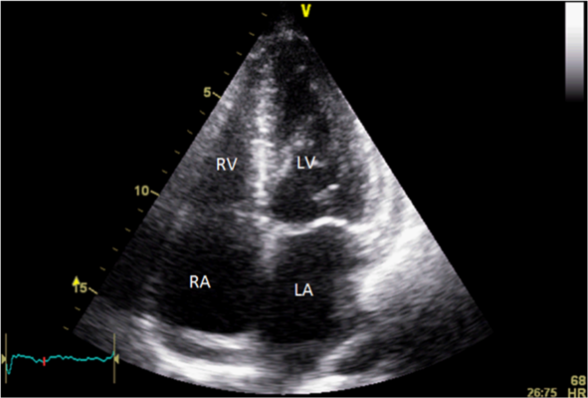
Is an apical four chamber view 2 weeks post pericardiocentesis and development of LV dysfunction showing resolution of the apical ballooning in systole with normal LV systolic function

**Fig. 8:**
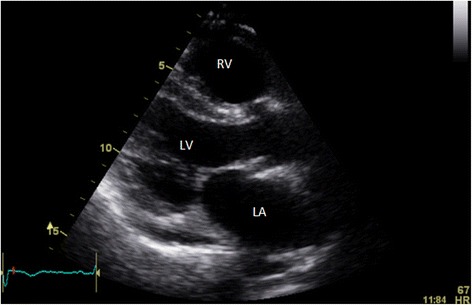
Parasternal long view in systole (2 weeks post pericardiocentesis and development of LV dysfunction), showing resolution of both akinesis in the mid septum and apical ballooning (apex not well visulised here)

**Fig. 9:**
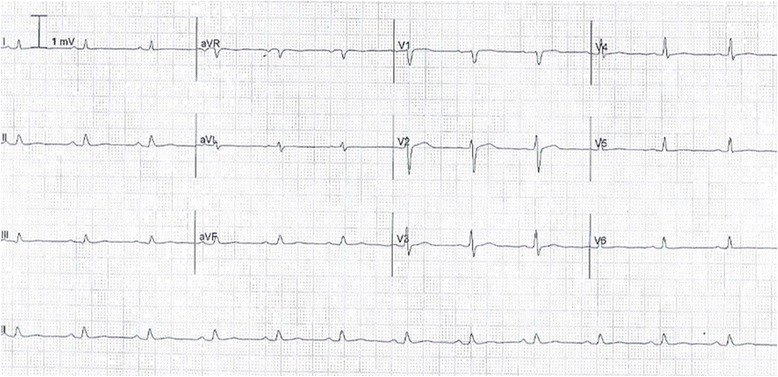
ECG 2 months post event demonstrating resolution of ischemic changes in ECG in Figure 6

Based on a presumptive diagnosis of SCM, angiotensin converting enzyme inhibitor and long acting beta-blocker were commenced, chemotherapy withheld and the patient discharged for early clinical and echocardiographic review. Serial follow up TTEs showed normalization of bi-ventricular function after two weeks (Figs. 7 and 8, Additional file 5: Video 5 and Additional file 6: Video 6), and restoration of R waves on subsequent ECGs (Fig. 9). Subsequent computed tomography examination showed normal coronary arteries with a calcium score of zero and no evidence of LAD laceration or dissection.

**Fig. 10:**
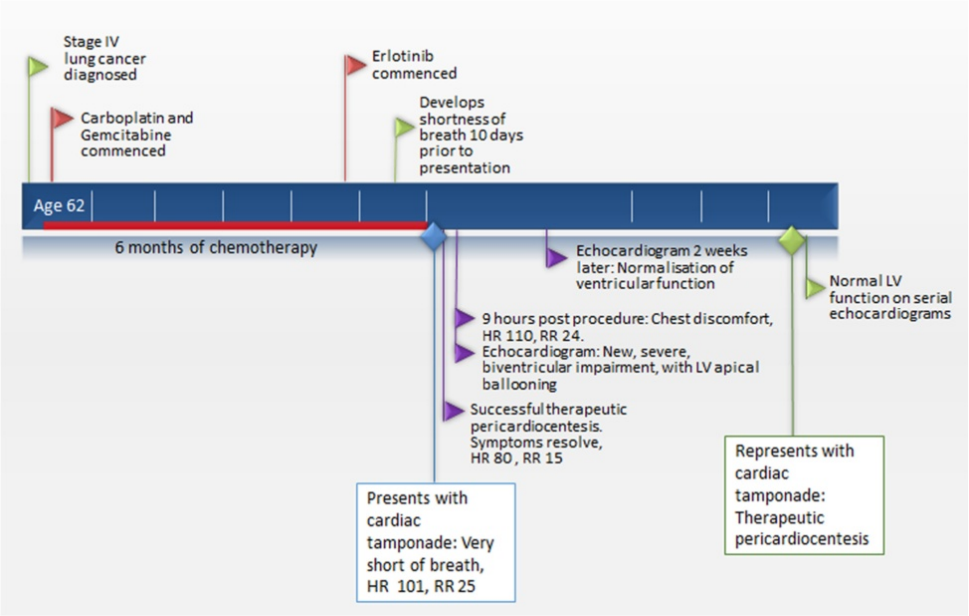
Time line of clinical events

The patient presented three months later with re-accumulation of pericardial effusion and tamponade. Therapeutic pericardiocentesis was performed with 500 ml of blood stained pericardial fluid drained immediately, with 1.9 L in total over 36 h. On this presentation he was relaxed and well adjusted in regards to his diagnosis. No LV dysfunction was detected on serial follow-up echocardiograms after the second pericardiocentesis (Fig. 10)

.

## Discussion

Our patient developed biventricular apical dysfunction following successful and judicious pericardiocentesis, with features typical of stress or “Takotsubo cardiomyopathy”. The case is instructive for its comparison with PDS and the clinical pattern of initial improvement followed by deterioration respectively due to pericardial aspiration and myocardial pathology.

In light of the timing of onset of biventricular impairment immediately post procedure PDS is an important differential diagnosis. Other differentials such as laceration to the ventricle or left anterior descending (LAD) coronary artery were clinically unlikely. The former was excluded by the absence of new pericardial bleed post procedure. Laceration of the LAD was also clinically unlikely given relatively small rise in cardiac enzymes and absence of large infarct, the presence of concurrent RV dysfunction, spontaneous recovery of ventricular function in a short period of time; additionally CT scan showed no evidence of haematoma or injury to the LAD.

Accordingly, we reviewed the literature describing SCM and PDS. Whereas SCM has been rarely reported after pericardiocentesis, much has been published on PDS. The incidence of PDS or new left or right systolic dysfunction has been reported to range from 5 % to 36 % of patients post pericardiocentesis [3, 4], especially after malignant pericardial effusions. Although the first case report of PDS in 1983 noted APO with preserved LV function [5], most subsequent reports describe severe impairment of left, right or bi- ventricular function, which may be segmental or global (Tables 1 and 2).

**Table 1:** Summary of reported cases of LVF post pericardiocentesis: Clinical characteristics

Report	Age/Gender	Clinical Scenario	Chronicity of effusion	Type of pericardi-ocentesis	Nature of pericardial fluid	Fluid drained	Time to onset of symptoms	Symptom	Signs
VanDyke (1983) [5]	42 M	Unwell for 10 days	Days	P	Exudate (malignant)	680 mls	Minutes	Dyspnoea	LVF
Shenoy (1984) [22]	57 M	Recent myocardial infarction	Days	P	Transudate	1000 mls	Minutes	Dyspnoea	LVF
Glasser (1988) [23]	33 M	Respiratory tract infection 3 months prior, history of Down’s and Ventricular Septal Defect	Weeks	S	Transudate	2000 mls	Minutes	Dyspnoea	LVF
Downey (1991) [24]	50 M	Traumatic (3 weeks post motor vehicle accident)	Weeks	P	Not specified	450 mls then 1500 mls	Minutes	Dyspnoea	LVF
Wolfe (1993) [19]	46 F	2 weeks, history of breast cancer prior	Weeks	P	Exudate	650 mls	Weeks	Dyspnoea	LVF
Wolfe (1993) [19]	50 F	2 weeks, history of breast cancer prior	Weeks	P	Exudate	650 mls	Weeks	Dyspnoea	LVF
Hamaya (1993) [25]	16 F	Unwell, lymphoma with pericardial effusion for 3 years	Months	P	Not specified	700 mls	Weeks	Dyspnoea	CS, and no APO
Braverman (1994) [26]	27 F	Unwell for 3 weeks (Atrial Septal Defect closure 13 years prior)	Weeks	P then S	Transudate	500 mls then 100 mls	Days	Dyspnoea, pleuritic chest pain	LVF, RVF, CS
Anguera (1996) [27]	68 F	History of bowel cancer, anorexia and dyspnoea for 1 month	Weeks	P	Malignant	800 mls	Minutes	-	CS
Sunday (1999) [8]	60 F	3 days of dyspnoea, lung cancer with pericardial involvement	Days	S	Exudate	700 mls	Minutes	Dyspnoea	CS, LVF
Chamoun (2003) [6]	36 F	2 months post Mitral valve replacement and Tricuspid repair	Days	P	Exudate	1070 mls	Hours	Dyspnoea	CS, LVF
Chamoun (2003) [6]	46 F	Metastatic cancer	Weeks	P	Exudate	1000 mls	Hours	Dyspnoea	CS, LVF
Geffroy (2004) [7]	53 M	1 month post chemotherapy for cancer	Weeks	S	Exudate	1500 mls	Not specified	Dyspnoea, hypoxia	CS, LVF, RVF
Ligero (2006) [20]	41 F	Lung cancer with hepatic metastases	Days	P	Exudate	1000 mls	Hours	Dyspnoea	LVF, RHF
Bernal (2007) [28]	45 F	Acute myeloid leukemia	Days	P	Exudate	500 mls	Hours	Dyspnoea	CS, LVF
Dosios (2007) [9]	66 F	Hematoma, 10 day history of dyspnoea	Days	S	Exudate	500 mls initially	Hours	-	CS
Sevimli (2008) [17]	42 F	Infective - tuberculous pericarditis	Days	S	Exudate	500 mls	Hours	Dyspnoea	CS and LVF
Khalili (2008) [29]	32 F	2 months post aortic and mitral valve replacement surgery	Weeks	P	Transudate	1000 mls	Hours	Dyspnoea	CS
Flores (2009) [30]	80 M	Unwell for weeks, multiple myeloma, stent 2 weeks prior	Weeks	P	Transudate	1200mls	Days	Dyspnoea	CS and LVF
Karamichalis (2009) [31]	19 F	2 months post motor vehicle accident	Weeks	P	Exudate	1600 mls	Hours	Dyspnoea	LVF
Lee (2010) [18]	14 M	Infective – tuberculous pericarditis	Days	P	Exudate	Not specified	Hours	Dyspnoea	CS, LVF
Lim (2011) [32]	44 F	Hypothyroidism related heart failure. Dyspnoea and fatigue for 4 months	Weeks	S	Exudate	1.3L	9 h	-	CS
Abdelsalam (2012) [10]	65 F	Stage IV Non small cell lung cancer for 6 months, 1 week of dyspnoea	Weeks	S	Malignant	Complete drainage of pericardial effusion intraoperatively	Seconds	Asystole during surgery	CS
Weijers (2013) [11]	69 F	Weight loss and dyspnoea	-	P	-	800 mls	6 h	-	LVF
Liang (2014) [1]	56 F	Polymyositis. Progressive dyspnoea on exertion	-	P	-	275 mls initially, with ongoing drain	Several hours	Pleuritic chest pain	Nil
Versaci (2015) [16]	78 F	3 months post mitral valve repair	Days	P	Possibly transudate	500 mls	Hours	Dyspnoea	LVF

*Abbreviations*: *P* percutaneous, *S* surgical, *CS* cardiogenic shock (hypotension, tachycardia), *LVF* Left heart failure, *RVF* right heart failure

**Table 2:** Summary of reported cases of LVF post pericardiocentesis: Electrocardiographic, biochemical, echocardiographic and outcome parameters

Report	LV function pre tap	LV function post tap	RV function post tap	Regional wall motion abnormality	Bio marker	ECG	Coronary artery imaging	Inotrope, IABP or Intubation	Death	LV recovery
VanDyke (1983) [5]	Normal	Normal (EF 67%)	-	Nil	Normal	Normal	-	Intubation	No	Normal LV
Shenoy (1984) [22]	-	Mild LV impairment	Normal	Septal hypokinesis	Normal	T wave abnormality and ST elevation V5-6	-	-	No	Normalised few days later
Glasser (1987) [23]	-	Pulmonary capillary wedge pressure normal	Normal (RVP increased)	-	-	-	-	Intubation	No	Clinical improvement
Downey (1991) [24]	-	Inferred to be normal	Normal	-	-	Normal	-	No	No	Normal LV
Wolfe (1993) [19]	Normal, EF > 50%	EF 30%	-	Severe global hypokinesis of LV	-	-	-	-	No	Normalised after 7 days
Wolfe (1993) [19]	Normal, EF > 50%	EF 25%	-	Antero-apical akinesis and apical dyskinesis	-	-	-	-	-	Normalised after 2 weeks
Hamaya (1993) [25]	Normal	-	-	Not provided	Normal	ST elevation	-	Inotropes and intubation	No	-
Braverman (1994) [26]	EF 20%	EF 20%	EF <15%	Not provided	-	-	-	-	-	EF 45% in 9 days then normalised after a few weeks
Anguera (1996) [27]	-	Mildly impaired. Normal capillary wedge pressure	Severely dilated and severely impaired contractility, EF <15%	Paradoxical septal motion	-	-	Normal coronary arteries	Inotropes	No	Complete recovery of biventricular fn after 10 days
Sunday (1999) [8]	EF 65%	EF 30%	Severely impaired contractility	Global hypokinesis	-	-	-	Intubation	Yes	No
Chamoun (2003) [6]	Normal, EF > 50%	EF 20%	-	Regional wall motion abnormality	-	SR	Normal coronary arteries	Inotropes and IABP	No	Normalised 2 weeks later
Chamoun (2003) [6]	Normal, EF > 50%	EF 20%	-	Akinesis of mid anterior wall and septum /dilatation of LV	-	SR	-	No	No	Normalised 2 weeks later
Geffroy (2004) [7]	Normal, EF > 50%	EF >50%	EF <15%	Akinetic and dilated RV	Elevated	Old RBBB	Normal coronary arteries	Inotropes and intubation	Yes	-
Ligero (2006) [20]	Normal, EF 75%	EF 25%	Severe impairment	Akinesis of anterior, septum and apex	Normal CK	Normal	Normal coronary arteries	Inotropes	No	Normalised 10 days later
Bernal (2007) [28]	Normal, EF 60-65%	EF 30%	-	Akinesis of mid anterior wall, anteroseptal akinesis with apical sparing	Elevated	Sinus tachycardia	CMR: no myocardial infarction	Inotropes and intubation	No	Normalised 1 weeks later
Dosios (2007) [9]	Normal LV fn	EF 25%	Moderately dilated, impaired	Global hypokinesis	Elevated	-	-	Inotropes and intubation	Yes	-
Sevimli (2008) [17]	Normal, EF > 50%	EF 20%	-	Akinesis in the left ventricular apex, and severe hypokinesis in the septum	-	Precordial TWI, normalised later	Normal coronary arteries	No	No	Normalised 10 days later
Khalili (2008) [27]	EF 35%	<10%	EF <15%	Global hypokinesis	-	Widening of QRS	-	Inotropes and IABP-	Yes	-
Flores (2009) [28]	EF 60%	13%	-	Global hypokinesis	Normal	Normal	Old RCA Branch lesion	Inotropes	No	Normalised 10 days later
Karamichalis (2009) [31]	-	-	-		-	Bradycardia	-	Inotropes and tracheostomy	Yes	-
Lee (2010) [18]	-	EF 20 -30%	-	Typical features of Takotsubo’s (diagnosed as such)	-	Precordial TWI, normalised later	Normal coronary arteries	No	Yes	No
Lim (2011) [32]	EF normal, 73%	EF 46%	-	Segmental wall motion abnormality	-	-	-	Inotropes and IABP	Yes	-
Abdelsalam (2012) [10]	Vigorous	EF 10-15%	Dilated and impaired fn	Takotsubo pattern of akinesia	-	ST elevation	-	Inotropes and IABP	Yes	-
Weijers (2013) [11]	Normal	Poor LV fn	-	General hypokinesia and anterior and septal akinesia	Normal	TWI and Q waves in anterolateral lead	-	-	No	Complete recovery of LV fn several months later
Liang (2014) [1]	Normal, EF 69%	EF 39% (on MRI)	Impaired	Severe mid and apical hypokinesis of both Ventricles (diagnosis : Takotsubo’s cardiomyopathy)	-	-	Normal coronary arteries	-	No	LV normalised 1 week later
Versaci (2015) [16]	Normal, EF >50%	EF 28%	-	LV ballooning, typical feature of Takotsubo’s cardiomyopathy	Elevated	QS wave in V1–V4 with negative T wave and ST elevation in V5–V6	Normal coronary arteries	No	No	Normalised after 10 days

*LV* Left ventricle, *RV* Right ventricle, *fn* function, *EF* Ejection fraction, *IABP* Intra-aortic balloon pump, *RVP* right ventricular pressure

A number of mechanisms have been proposed to explain the pathogenesis of LV systolic dysfunction in PDS. Acute withdrawal of exaggerated sympathetic drive during relief of tamponade may trigger paradoxical haemodynamic instability [5]. Mechanical, inter-ventricular volume mismatch may also contribute, with sudden relief of pericardial constraint leading to abrupt, disproportionate increase in RV volume and a paradoxical rise in pulmonary artery pressure, resulting in raised LV end diastolic pressure and transient left heart failure [5–9]. Others have proposed myocardial stunning from coronary perfusion mismatch with acute distension of cardiac chambers after decompression [6, 10, 11]. Taken together, it is likely that a combination of hormonal and mechanical pathophysiologic mechanisms contribute to LV dysfunction and the final clinical sequelae in PDS.

The classic echocardiographic feature in SCM is transient LV apical ballooning, although other segmental patterns have been described [12, 13]. A stressor leading to sympathetic overdrive and excessive catecholamine release is the currently accepted trigger in the development of SCM [12]. The catecholamine surge precipitates 1) ‘peripheral arterial vasospasm leading to increased afterload and transient increase in LV end-systolic pressure’, 2) ‘acute multiple coronary artery vasospasm leading to myocardial ischaemia’, and 3) direct catecholamine- β-adrenoceptor - mediated myocardial stunning in the apex [14]. These three pathophysiologic pathways are thought to contribute to the ischaemia, morphologic features and potential haemodynamic sequelae that can be seen in SCM.

More recent case reports have made reference to LV apical ballooning related to PDS as similar to SCM [10, 11, 15–17], and have postulated the physiological stressor being cardiac tamponade along with emotional stress [16]. It is therefore possible that the transient ventricular systolic dysfunction in PDS is actually a variant form of stress cardiomyopathy. We carefully reviewed 25 cases of heart failure post pericardiocentesis in the literature (Tables 1 and 2), and we believe that seven cases (two considered to be SCM [1, 18] by the authors and five classified as PDS [10, 16, 17, 19, 20]) could be considered to have echocardiographic features of SCM.

SCM has relatively characteristic clinical presentation, with rise of cardiac enzymes [21], and often associated with ischaemic ECG changes (up to 44 % of those with SCM have T-wave inversion and 41 % ST elevation [13, 21]). The clinical manifestations in PDS are more variable, ranging from asymptomatic in some to severe low cardiac output states in others. The primary clinical symptom in PDS has been reported as dyspnoea (Table 1). This is in contrast to chest pain being predominant in SCM (69-83 % of presentations) [13, 21]. In the majority of cases of PDS in the literature (Table 2) there was no cardiac enzyme rise, and ischaemic type changes on ECG were seen in a minority (seven of twenty five cases). In all the cases where ischaemic ECG changes where present except for one, there was concomitant apical and peri-apical regional wall motion abnormality, which could be classified as SCM also.

Generally SCM has a benign course, with recovery of LV function and good prognosis [12], whilst PDS has poorer outcomes and increased mortality [4]. Reports of PDS suggested normalization of LV dysfunction in 12 of 25 cases classified as PDS. Of the 12 cases that did recover LV function, four had LV impairment with classic SCM pattern of LV impairment on echocardiogram [16, 17, 19, 20]. The normalisation of LV function in our patient 2 weeks subsequently is more in keeping with SCM.

Current literature has not specifically addressed risk factors for the development of ventricular dysfunction after pericardiocentesis. In our patient, the malignant nature of the effusion, the presence of tamponade and larger size of pericardial effusion [4], may have increased his predisposition to develop ventricular dysfunction. Amount and rate of fluid removed on initial decompression are also associated with development PDS [4, 5], however there are no guidelines regarding the maximum amount of pericardial fluid that can be drained immediately. There is consensus to stop initial drainage with improvement of symptoms or hemodynamic parameters, followed by gradual decompression through indwelling catheter [5].

Our patient’s apical systolic dysfunction post pericardiocentesis was associated with chest discomfort, transient loss of R waves and rise in cardiac enzymes are typical of classic SCM. The clinical sequence of HR and RR improving immediately post decompression and then increasing again hours after the procedure, was a useful clinical marker of myocardial dysfunction, prompting investigation which identified new ventricular impairment. It is likely that the frequency of transient LV dysfunction is underestimated in these patients.

## Conclusion

We report a case of transient biventricular dysfunction post pericardiocentesis, with classic features of SCM. LV dysfunction post pericardiocentesis and in PDS is more prevalent than previously thought, and some previous reports of PDS may also be potentially considered as SCM complicating pericardiocentesis. In addition to judicious and gradual decompression to avoid ventricular dysfunction or PDS, patients undergoing therapeutic pericardiocentesis should have careful haemodynamic monitoring, as changes in parameters such as heart rate and respiratory rate can raise suspicion of acute LV impairment.

### Consent

Written informed consent was unable to be obtained from the patient for publication of this Case report and any accompanying images, as he has passed away. His next of kin are not contactable after their subsequent return to their home country of China. Professor L. Kritharides, Head of Department, approves the publication of this report, with all patient identifiers kept confidential and material presented solely for educational purposes arising from the clinical encounter.

## Additional files

Additional file 1: Video 1. Apical four chamber view demonstrating large pericardial effusion on presentation, with tamponade and diastolic compression of right heart chambers. Note the LV function is normal. Additional file 2: Video 2. Parasternal long view demonstrating large pericardial effusion on presentation, with tamponade and diastolic compression of right heart chambers. Note the LV function is normal. Additional file 3: Video 3. Apical four chamber view performed on the following morning after the patient experienced chest discomfort. The main finding is severe biventricular impairment, with akinesis of the apex and periapical areas. There is small residual pericardial effusion. Additional file 4: Video 4. Parasternal long view performed on the following morning after the patient experienced chest discomfort. The main finding is severe biventricular impairment, with akinesis of the apex and periapical areas. There is small residual pericardial effusion. Additional file 5: Video 5. Apical four chamber view performed nearly 2 weeks after the pericardiocentesis and subsequent stress cardiomyopathy. They show normalisation of both left and right ventricular function. Additional file 6: Video 6. Parasternal long view performed nearly 2 weeks after the pericardiocentesis and subsequent stress cardiomyopathy. They show normalisation of both left and right ventricular function.

## Abbreviations

SCM: Stress Cardiomyopathy
LV: Left ventricle
RV: Right ventricle
PDS: Pericardial decompression syndrome
APO: Acute pulmonary oedema
ECG: Electrocardiogram
TTE: Transthoracic echocardiography
bpm: Beats per minute
HR: Heart rate
RR: Respiratory rate
